# Human papillomavirus and prostate cancer: systematic review and meta-analysis

**DOI:** 10.1038/s41598-023-43767-7

**Published:** 2023-10-03

**Authors:** Irina A. Tsydenova, Marina K. Ibragimova, Matvey M. Tsyganov, Nikolai V. Litviakov

**Affiliations:** 1 Cancer Research Institute, Tomsk National Research Medical Center of the Russian Academy of Sciences, Tomsk, Russia 634028; 2 National Research Tomsk State University, Tomsk, Russia 634050; 3 Siberian State Medical University, Tomsk, Russia 634050

**Keywords:** Oncology, Risk factors, Prostate cancer

## Abstract

The involvement of human papillomavirus (HPV) in the prostate carcinogenesis is a controversial issue. The presented meta-analysis was carried out to systematize the currently available research results regarding this question. The meta-analysis includes case–control studies from 1991 to 2022, which were collected from publicly available bibliometric databases. The meta-analysis was performed using Meta-Essentials_1.5 software. We used Begg’s and Egger’s methods to assess publication bias. Cochran’s *Q* test was used to assess heterogeneity and the *I*^2^ index was employed for calculating the variation in the pooled estimations. The analysis was based on data from 27 case–control studies, which in total yielded 1607 tumour tissue samples of prostate and 1515 control samples (317 samples of normal tissue, 1198 samples of benign prostatic hyperplasia (BPH)). According to the data obtained, there was high risk of prostate cancer by HPV infection in both cases. HPV was found in prostate cancer in 25.8% of cases, while in normal tissue samples the virus was detected in 9.2% of cases and in 17.4% with BPH as a control. In particular, more studies on the association of HPV and prostate cancer are needed to prove the role of HPV in the development of prostate cancer. In addition to the controversial question of whether HPV infection is associated with prostate cancer risk, it is worth considering whether the samples used as a control have an impact on the results. The impact of HPV in prostate tumour tissue samples on outcome should also be investigated.

## Introduction

Prostate cancer is a major public health problem, being the most common malignancy of solid organs in men worldwide^1^. According to GLOBOCAN estimates, prostate cancer is the second most common cancer, the fifth leading cause of death among men, and the most frequently diagnosed cancer in more than half of the countries in the world. The highest incidence rates are observed in Northern and Western Europe, the Caribbean, Australia/New Zealand, North America and South Africa, while the lowest rates are in Asia and North Africa^2^. Such variability in morbidity and mortality in different countries can be explained not only by socioeconomic status and different health policies, but also by the prevalence of prostate-specific antigen screening, which is used to detect indolent cases in a particular country^3^. Despite the fact that in Russia the incidence of prostate cancer is at a relatively low level compared to other countries, the mortality rate ranks second after lung cancer^2^.

Relatively little is known about the etiology of this common disease. The established risk factors are limited to age, family history, and genetic predisposition. Up to 20% of men with prostate cancer have a family history of the disease^4^. The presence of any affected family member approximately doubles the risk of cancer^5^. According to a study by Grill et al., having one first-degree relative younger than 60 years of age at the time of diagnosis increases the risk of prostate cancer by 2.5 times (1.6 times for first-degree relatives aged ≥ 60 years)^6^. The genetics underlying family predisposition and hereditary cancer syndromes associated with prostate cancer are complex. Nyberg et al. identified one high-risk gene specific for prostate cancer in which men with an inherited *HOXB13* mutation were at increased risk of prostate cancer throughout their lives^7^. There are also data in the literature about other genes that are responsible for predisposition to prostate cancer, such as *BRCA1*, *BRCA2* and the *MSH2* gene associated with Lynch syndrome^8,9^. Ethnicity also plays an important role in the prostate cancer etiology. Black men in the United States and the Caribbean have the highest incidence rate in the world, while Asian men, according to the literature, have the lowest risk of developing prostate cancer^10–13^. Recent studies have shown that genetic predisposition may be a potential risk factor for prostate cancer in men of African descent^14,15^.

Only 10–15% of all human cancers are caused by viruses^16^. These viruses modify tumour cell behavior by inhibiting tumour suppressor signaling, blocking apoptosis pathways, promoting metastasis and triggering angiogenesis. Human papillomavirus is one particularly important oncogenic virus. We have previously carried out 2 meta-analyses that assessed the association of HPV infection with endometrial and ovarian cancer. Our previous meta-analysis did not find an association of HPV infection with the risk of endometrial cancer. It has been suggested that the reason for the low persistence of HPV in endometrial cancer and the lack of association of the virus with the risk of this type of cancer is due to the deeper layer of the endometrium, as it is involved in the repair of the uterine mucosa after rejection of the surface layer^17^. Analysis of the association of HPV with the risk of ovarian cancer showed that HPV was found in 28.6% of cases of ovarian cancer, while in the control group samples the virus was detected in 8.8%. However, a geographical heterogeneity was found in the prevalence of HPV in ovarian cancer, with a higher risk in those from the Asian continent^18^. According to the literature, HPV, which is best known for its ability to cause cervical cancer in women, may be associated with the prostate cancer pathogenesis. Evidence from genetic and clinical studies suggests that there may be a causal relationship between prostate cancer and human papillomavirus^1^. For example, Whitaker et al. concluded in their study that the identification of HPV-associated koilocytes in prostate cancer samples is an indication of HPV infection and the potential oncogenic effect of the virus on prostate cancer^19^. Medel-Flores et al. obtained significant results of HPV and prostate cancer association; they also found koilocytes in all in situ HPV-PCR-positive specimens^20^. Also interesting is a study by Glenn et al. in which authors showed that high-risk HPV is present in benign prostate tissues prior to the development of HPV-positive prostate cancer. Their study observed higher expression of HPV E7 oncoproteins in benign prostate tissues compared to advanced prostate cancer that subsequently developed in the same patients. Their observation suggests that HPV oncogenic activity is an early phenomenon in most prostate oncogenesis^21^. But, in addition to studies that have found an association between HPV and prostate cancer, there are others that have not confirmed a role for HPV infection in prostate carcinoma^22,23^. Nahand et al. didn’t find significant correlation between the presence of HPV and prostate cancer, but they observed a significant relationship between the viral proteins expression and cellular factors involved in the malignant progression of prostate cancer. The authors concluded that it is likely that HPV infection potentially modulates prostate tumour cell behavior by influencing inflammation, angiogenesis, apoptosis mechanisms, and suppression of antitumour immunity, which in turn contributes to oncogenesis^24^.

From the above, we can conclude that the presented scientific results are highly controversial contradictory and require systematization. Thus, our aim is to systematize the available data regarding HPV and prostate cancer, which will help in deciding on the prospects for further research on the association of HPV with this type of cancer.

## Methods

All included studies were case–control studies. Tumour prostate tissue samples were the mandatory study material.

### Research selection criteria

The following modified participants, interventions, comparators, outcomes, and studies approach (PICOs) guided eligibility screening of studies for inclusion in our study (Supplementary Material—S1 Appendix). Mandatory criteria for inclusion in the meta-analysis and the systematic review were:

1. Strict consistency with localisation and diagnosis. Diagnoses must be confirmed morphologically.
2. The presence and type of HPV must be determined by PCR only.
3. PCR examination of normal tissue control samples is mandatory.
4. Tissue or paraffin blocks or frozen tissue must be used.

Exclusion criteria: serum or other test material; no control; no comparison of prostate tumour tissue with a control of normal prostate tissue or benign prostatic hyperplasia; any HPV detection method other than PCR.

### Literature search strategy and selection process

**Figure 1:**
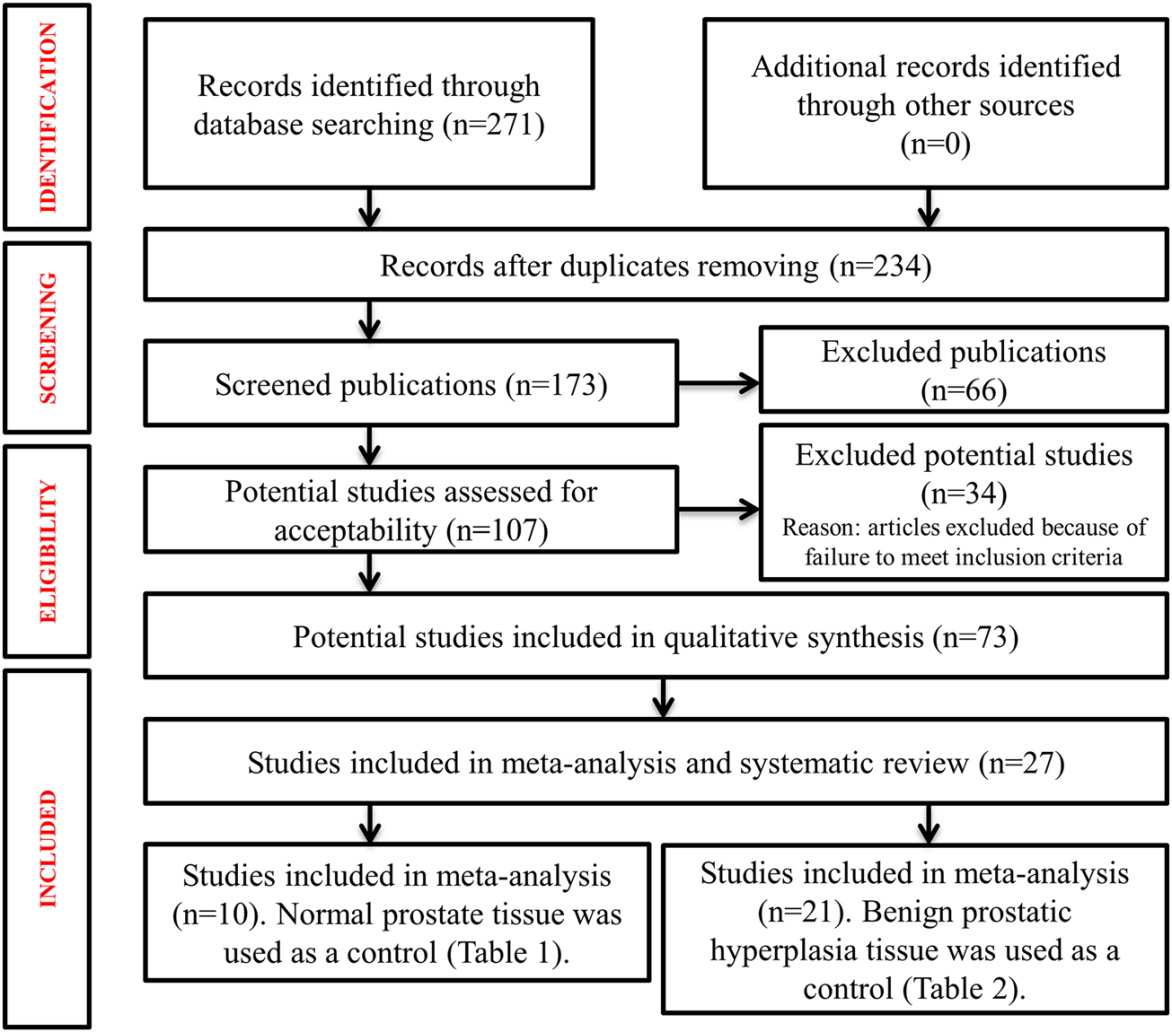
Flowchart of included studies.

I.A.T. and M.K.I. searched 4 health, social care databases (PubMed, Embase, Scopus and Medline) to August 21, 2022. We also carried out a ‘snowball’ search to identify additional studies by searching the reference lists of publications eligible for full-text review and using Google Scholar to identify and screen studies citing them (Fig. 1). The search strategy included a combination of indexing terms as well as keyword terms including “Prostate cancer”, “Human papillomavirus”, “Risk factors”, “association of HPV with cancer”, “HPV and BPH”. We excluded articles that did not meet the eligibility criteria (Supplementary Material—S1 Table). The search strategy is included in (Supplementary Material—S2 Table).

### Data extraction

We independently reviewed titles and abstracts of the first records and discussed inconsistencies until consensus was obtained. If necessary, N.V.L. was consulted to make the final decision. I.A.T. and M.K.I. extracted all relevant information from the studies, including: author, publication date, number of HPV positive and negative patients, HPV subtypes.

### Quality assessment

Risk of bias was evaluated with a Joanna Briggs Institute (JBI) checklist for case control studies (Supplementary Material—S3 Table). Data were obtained independently by authors. Based on this scale studies with a quality score of 7 to 10 were categorized as very good/good, a score of 5 to 6 was categorized as having satisfactory quality, and a score less than 5 was taken as unsatisfactory quality.

The GRADE (Grading of Recommendations, Assessment, Development, and Evaluations) tool was used to assess the quality of evidence for the primary outcome. The assessment was based on five parameters: risk of bias, inconsistency (or heterogeneity) between studies, indirectness, imprecision (risk of random errors), and publication bias. The evidence for each item was rated as high, moderate, low, or very low (Supplementary Material—S4 Table)^25^.

### Statistical analysis

Two meta-analyses were carried out that differed between samples from the control group. In 1 case, normal prostate tissue samples were used as the control group and in 2 case, benign prostatic hyperplasia samples were used as the control group. The meta-analysis was performed using Meta-Essentials_1.5 software^26^. We used Begg’s and Egger’s methods to assess publication bias. The funnel plots shape is used to show symmetry, and Egger’s methods are applied to indicate significant publication bias for the analysis exploring association between risk of prostate cancer and HPV. Cochran’s *Q* test was used to assess heterogeneity and the *I*^2^ index was employed for calculating the variation in the pooled estimations. All *p* values are two-tailed, *α* ≤ 0.05 was considered statistically significant (*p* ≤ 0.05).

## Results

The meta-analysis includes case–control studies from 1991 to 2022, which were collected from publicly available bibliometric databases. We found 271 records in databases searching. After duplicates removal, 234 records were screened, from which we reviewed 73 full-text documents, and finally included 27 articles. These 27 case–control studies include 1607 prostate tumour tissue samples and 1443 control samples (413 normal tissue, 1308 BPH) (Fig. 1). The studies in chronological order collected for this meta-analysis are presented in Tables 1 and 2. Tumour prostate tissue samples were the mandatory study material. In Table 1 normal prostate tissue was used as a control, in Table 2 samples of benign prostatic hyperplasia was taken as a control.

**Table 1:** Prevalence of HPV in prostate cancer with normal tissue as a control.

Author (Year), Country	Genotype of HPV	Tumour tissue		Controls		p-value
		Total	HPV +	Total	HPV +	
McNicol and Dodd (1991), Canada^27^	16, 18	27	14 (52%)	5	1 (20%)	0.338
Ibrahim et al. (1992), US^28^	6, 11, 16, 18, 31, 33, 35	24	3 (13%)	20	2 (10%)	1
Suzuki et al. (1996), Japan^29^	16	51	8 (16%)	51	0 (0%)	0.006
Terris and Peehl (1997), USA^30^	16	53	10 (19%)	37	5 (14%)	0.576
Martinez-Fierro et al. (2010), Mexico^31^	33, 45, 52, 58, 66, 68, 83, 44, 81	55	11 (20%)	75	4 (5%)	0.012
Whitaker et al. (2013), Australia^20^	18	10	7 (70%)	10	1 (10%)	0.02
Michopoulou et al. (2014), Greece^32^	16, 18, 31	50	8 (16%)	30	1 (3%)	0.145
Smelov et al. (2016), Russia^33^		13	7 (54%)	13	4 (31%)	0.428
Nahand et al. (2020), Iran^24^	2, 6, 16, 18, 33	58	19 (33%)	32	5 (16%)	0,088
Fatemipour et al. (2021), Iran^34^	2, 6, 11, 16, 18, 33	72	26 (36%)	44	7 (16%)	0.021
Total		413	114 (28%)	317	30 (10%)	0.0000012

**Table 2:** Prevalence of HPV in prostate cancer with benign prostatic hyperplasia as a control.

Author (Year), Country	Genotype of HPV	Tumour tissue		Controls		p-value
		Total	HPV+	Total	HPV+	
McNicol and Dodd (1991), Canada^27^	16, 18	27	14 (52%)	56	34 (61%)	0.483
Ibrahim et al. (1992), US^28^	6, 11, 16, 18, 31, 33, 35	24	3 (13%)	16	0 (0%)	0.261
Rotola et al. (1992), Italy^35^	6, 11, 16	8	6 (75%)	17	14 (82%)	1
Moyret-Lalle et al. (1995), France^36^	16, 18	17	9 (53%)	22	7 (32%)	0.107
Terris and Peehl (1997), USA^30^	16	53	10 (19%)	21	7 (33%)	0.224
Noda et al. (1998), Japan^37^	16	38	0 (0%)	71	3 (4%)	0.324
Serth et al. (1999), Germany^38^	16	47	10 (21%)	37	1 (3%)	0.019
Carozzi et al. (2004), Italy^39^	6, 11, 16, 18, 31, 33, 35, 45, 52, 58	26	17 (65%)	25	12 (48%)	0.264
Leiros et al. (2005), Argentina^40^	11, 16	41	17 (41%)	30	0 (0%)	0.00003
Bergh et al. (2007), Sweden^41^	–	201	0 (0%)	201	0 (0%)	1
Silverstre et al. (2009), Brazil^22^	16, 84	65	2 (3%)	6	0 (0%)	1
Chen et al. (2011), Australia^42^	18	51	7 (14%)	11	3 (27%)	0.363
Aghakhani et al. (2011), Iran^23^	16, 18, 6, 11	104	13 (13%)	104	8 (8%)	0.358
Tachezy et al. (2012), Czech Republic^43^	6, 11, 16, 18, 31, 33	51	1 (2%)	80	2 (3%)	1
Salehi and Hadavi (2012), Iran^44^	–	68	3 (4%)	85	0 (0%)	0.086
Whitaker et al. (2013), Australia^20^	18	10	7 (70%)	10	2 (20%)	0.07
Singh et al. (2015), India^45^	6, 11, 16, 18	95	39 (41%)	55	11 (20%)	0.011
Huang et al. (2016), China^46^	16, 18	75	30 (40%)	73	9 (12%)	0.001
Aydin et al. (2017), Turkey^47^	57	60	1 (2%)	36	0 (0%)	1
Abdolmaleki et al. (2018), Iran^48^	18	58	2 (3%)	75	1 (1%)	0.41
Medel-Flores et al. (2018), Mexico^19^	6, 11, 16, 18, 31, 33, 52, 58	189	37 (20%)	167	16 (10%)	0.016
Total		1308	228 (17%)	1198	130 (11%)	0.220

The meta-analysis included 27 case–control studies using PCR technology. Of these, 6 studies where normal prostate tissue was used as a control (Table 1), 17 studies where samples from benign prostatic hyperplasia were used as a control (Table 2), and 4 studies that assessed both prostate tissue and benign prostatic hyperplasia as controls (Tables 1, 2).

**Figure 2:**
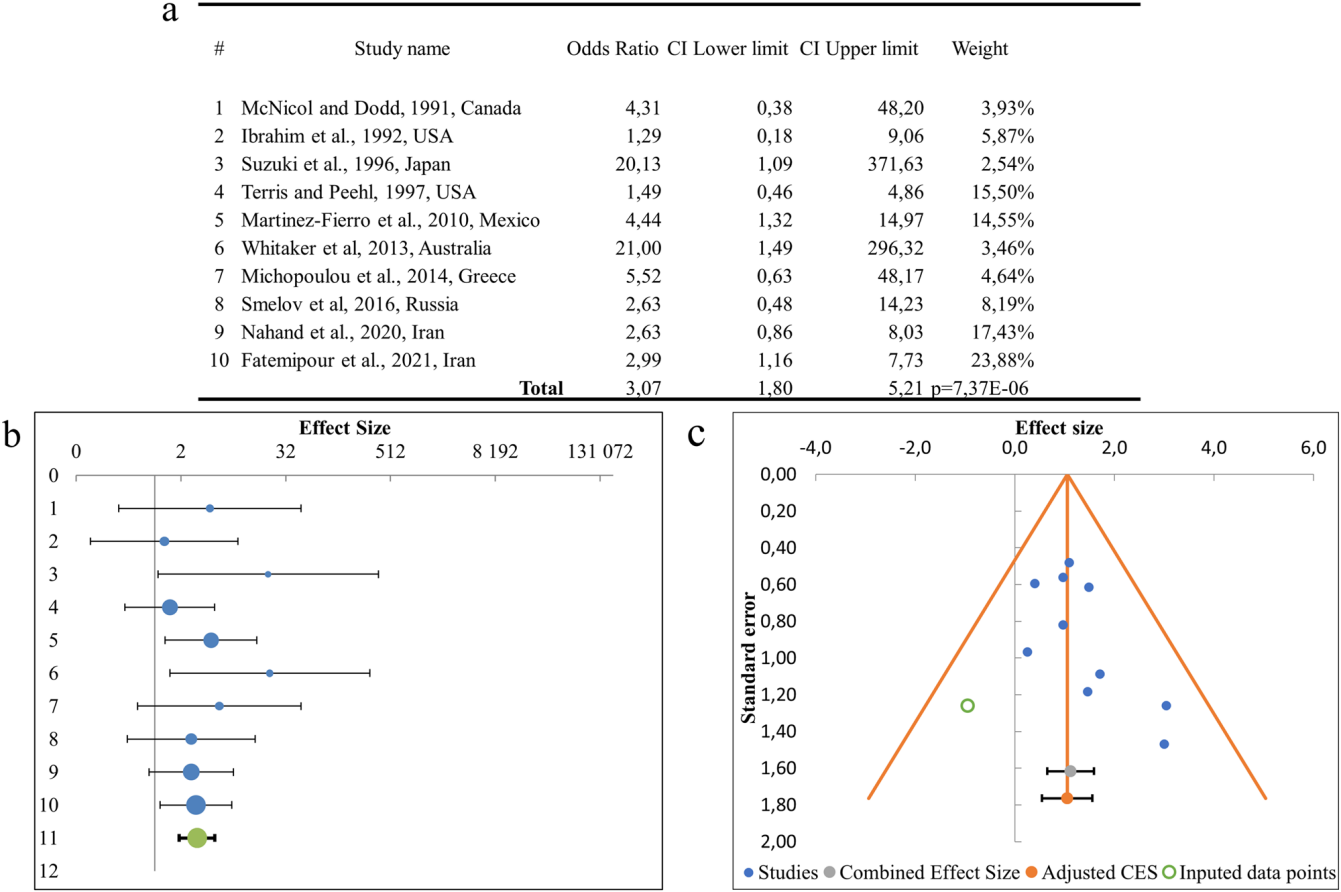
Association between HPV infection and prostate cancer risk. (**a**) Results of the meta-analysis of the association between HPV infection and prostate cancer risk; (**b**) odds ratio in men positive for HPV; (**c**) Begg’s funnel plot of studies investigating HPV as a risk factor.

The result of the meta-analysis of the 10 studies comparing prostate cancer to normal tissue is shown in Fig. 2. Analysis of the data on the prevalence of HPV according to the studies included in the meta-analysis showed that HPV is found in prostate cancer in 25.8% of cases, while in normal tissue samples the virus was detected in 9.2% of cases.

The cumulative odds ratio OR (OR 95 CI) of prostate cancer risk in HPV infection was 3.07 (1.80–5.21) (wherein relative risk RR (RR 95 CI) 1.17 (1.09–1.26), p = 0.0000012), the differences were statistically significant and p = 7.37e−6. The sample heterogeneity index *I*^2^ = 0% and the Cochrane Q-test was p = 0.626, at the required p < 0.1 level, so a Random model was used. A funnel plot was created to assess publication bias using Egger’s funnel plots. This graph shows no significant asymmetry (Fig. 2B,C) and according to Begg’s test there was no significant systematic publication bias in this meta-analysis (p = 0.089). However, the result of the Egger’s test was not significant (p = 0.097).

**Figure 3:**
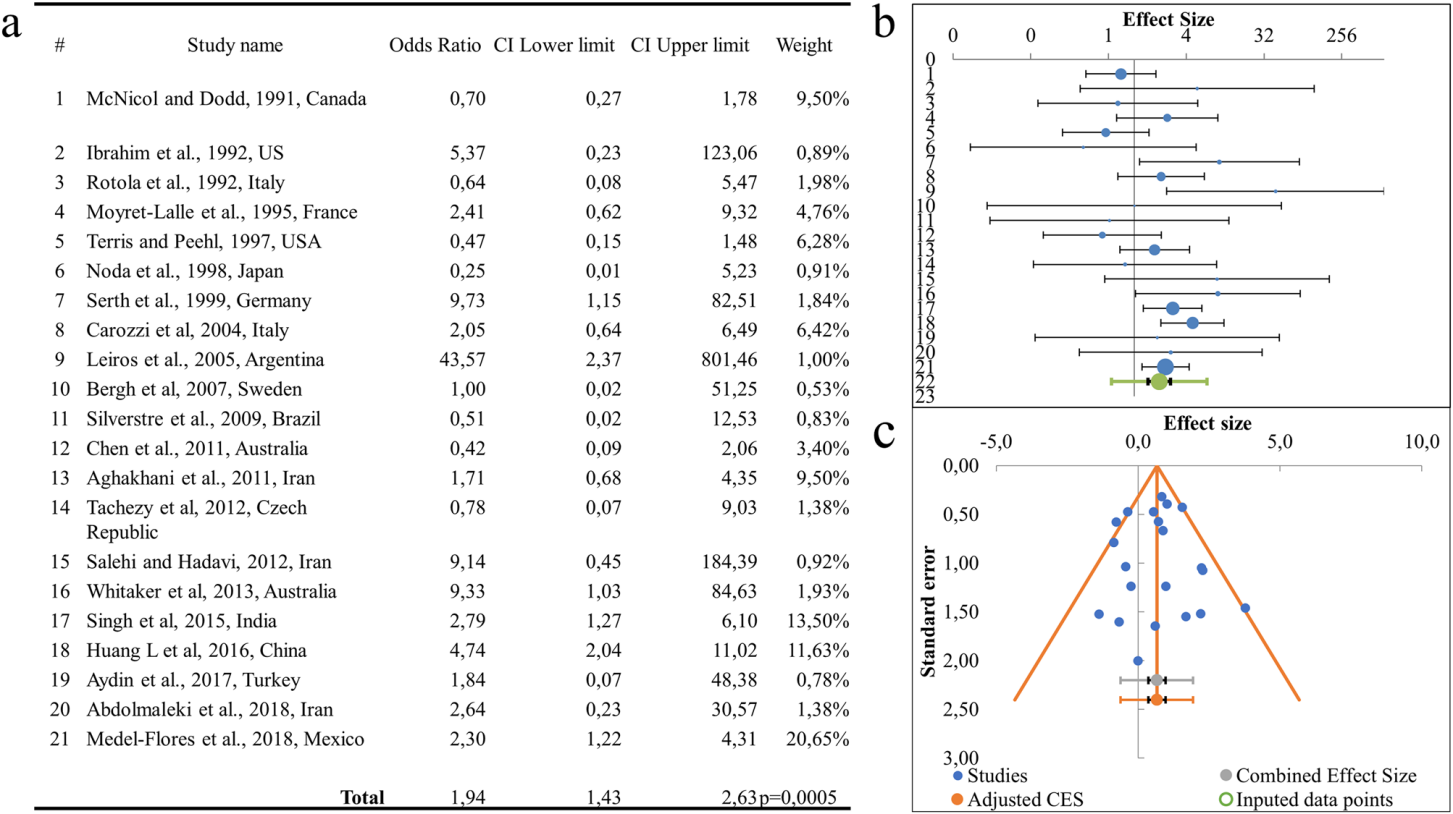
Association between HPV infection and prostate cancer risk, relative to pre-tumour prostate pathology. (**a**) Results of the meta-analysis of the association between HPV infection and prostate cancer risk; (**b**) odds ratio in men positive for HPV; (**c**) Begg’s funnel plot of studies investigating HPV as a risk factor.

The meta-analysis result of 21 studies comparing prostate cancer to benign prostatic hyperplasia is presented in Fig. 3. Analysis of data on the prevalence of HPV according to the studies included in the presented meta-analysis showed that HPV is found in 17.4% of cases of prostate cancer, while in the pre-tumour pathology samples the virus was detected in 10.8% of cases.

The cumulative odds ratio OR (OR 95 CI) of prostate cancer risk in HPV infection was 1.94 (1.43–2.63) (wherein relative risk RR (RR 95 CI) 1.01 (1.00–1.02), p = 0.220), the differences were statistically significant and p = 0.0005. The sample heterogeneity index was *I*^2^ = 42.77% and Cochrane Q-test was p = 0.020, at the required p < 0.1, so a Fixed model was used. The funnel plot shows no significant asymmetry (Fig. 3B,C), and according to the Begg’s test, there was no significant systematic publication error in this meta-analysis (p = 0.856). However, the result of the Egger’s test was not significant (p = 0.956).

According to the available literature data, the frequency of HPV prevalence in benign prostatic hyperplasia (BPH) ranges from 0 to 65%^41^. The most frequently determined HPV types in the presented studies were 16, 18, 31, 33 virus types^49^. However, it is interesting to note that in a 2018 study, Medel-Flores et al. showed that HPV 52 and 58 were the most frequent genotypes (33% and 17%, respectively)^19^.

## Discussion

Based on the meta-analysis results, we can conclude that the risk of prostate cancer by HPV infection is high in both cases OR 3.07, 95% CI 1.80–5.21 if normal prostate tissue is taken as a control and OR 1.94, 95% CI 1.43–2.63 if BPH is taken as a control. However, in meta-analysis using a risk ratio, a very different picture is observed. The association of HPV and prostate cancer risk is significant in the studies when normal prostate tissue is used as a control (RR = 1.01, 95% CI 1.09–1.26, p = 0.0000012) compared to studies when BPH is used as a control (RR = 1.01, 95% CI 1.00–1.02, p = 0.220). When using BPH as a control, HPV was detected in 10.8% of pre-tumour samples, whereas in normal tissue samples it was detected in 9.2% of cases. The HPV prevalence in prostate cancer varies up to 25.8% with normal prostate tissue as a control and up to 17.4% with BPH as a control. In addition to the controversial question of whether HPV infection is associated with prostate cancer risk, it is worth considering whether the samples used as a control have an impact on the results.

The first research data on the association of human papillomavirus with prostate cancer appeared as early as 1990, when McNicol and Dodd identified HPV types 16 and 18 in prostate tumour tissue. Since then, an intensive study of the HPV prevalence in prostate cancer began. In a 2017 meta-analysis, the authors draw attention to the fact that most studies use BPH as a control^50^. In their opinion, the results may differ depending on where the tissue sample was taken. However, judging from the studies in this meta-analysis, where both normal prostate tissue and BPH were taken as controls^27,28,30,37^, it can be concluded that with a clear quantitative advantage of BPH samples compared to normal tissue, the HPV detection percentage in BPH is higher. No significant association between HPV and BPH was found in these studies. According to the literature, many past authors expressed the opinion that HPV probably doesn’t play a causal role in the prostate cancer development because there is an equal prevalence of high-risk HPV in benign and malignant prostate tissues^21,24,34,51^. One reason for the low HPV definition may be that the prostate gland, as a derivative of the urethra, is composed of half glandular cells, 1/4 fibrous and 1/4 muscular fibres. However, it is still unclear whether HPV infection can cause morphological changes in the glandular epithelium^17^. It is also interesting to note that DNA detection could determine the current infection status only if the pathogen had infected the tissue^50^. Because there is a “hit and run” phenomenon of HPV, previously described by other researchers, in which HPV-infected cells temporarily acquire a complete or incomplete viral genome in the early stages of cancer development, but the virus becomes undetectable in the later stages of cancer^21^. This contrasts with the causal role of HPV in the development of cervical cancer, in which HPV is necessary for both initiation and maintenance of tumourigenesis. This obvious involvement of HPV in the early stage of prostate tumourigenesis could explain the immensely viral load of HPV in fully developed prostate cancer^50,52^.

It is also interesting to note the results of a retrospective cohort study where the authors showed that high-risk HPVs, predominantly HPV 16 and 18, are typically present in benign prostate tissues 1–10 years before developing HPV-positive prostate cancer. Similar results were obtained in another meta-analysis; HPV-16 infection was significantly associated with an increased risk of prostate cancer (OR 1.38), whereas HPV-18 was not (OR 1.05)^51^. There is a higher prevalence of HPV E7 oncoprotein expression in benign prostate tissues compared to subsequent prostate cancer specimens from the same patient. Thus, the much higher prevalence of HPV oncoprotein E7 expression in benign prostate tissues compared to subsequent prostate cancer in the same patients suggests that HPV oncogenic activity is an early phenomenon in prostate tumourigenesis^21^. Furthermore, one of the key markers of tumour progression is the determination of HPV genome physical forms. Khatami et al. in their study, besides detecting HPV prevalence, determined the physical status of the HPV genome in HPV-positive samples and found that 50% of samples had HPV in an integrated form, which is a key event in the malignization process. In addition, the integrated HPV DNA is characterized by overexpression of E6 and E7 genes, as the break of the ring DNA occurs in the localization area of E1 and E2 genes. The authors also concluded that HPV infection may play a role in tumour progression by enhancing resistance to apoptosis in human prostate tumour cells^52^. In cervical cancer and HSIL, the integrated form of HPV occurs much less frequently, in 26.5% and 22.9% of cases, respectively, but also plays a negative role in the survival of cervical cancer patients and the risk of neoplasia malignization^53,54^. Because of the possible role of HPV in prostate cancer, it can be argued that it is becoming increasingly important to intensify existing HPV vaccination strategies. Studies based on high carcinogenic risk HPV typing and virological indicators such as viral load and viral physical status are needed to further analyse the association of HPV with prostate cancer risk and its impact on disease prognosis.

## Conclusion

Despite the heterogeneity of the current literature on methods for detecting HPV in patients with prostate cancer, HPV infection should be considered as a potential risk factor for the development of prostate cancer. The present meta-analysis shows a significant prevalence of HPV infection in prostate tumour tissue samples and the association of HPV with prostate cancer when normal prostate tissue was used as a control. This makes further studies in this direction promising. In particular, more studies on the association of HPV and prostate cancer are needed to prove the role of HPV in the development of prostate cancer. This kind of study should be carried out with the inclusion of normal prostate tissue as a control instead of BPH. There is also a need to investigate the effect of HPV in the prostate tumour tissue samples on the outcome of the disease.

### Supplementary Information

Supplementary Information.

## Data Availability

The datasets used and/or analysed during the current study available from the corresponding author on reasonable request.
