# Supplementary material for: Human papillomavirus and prostate cancer: systematic review and meta-analysis
Source: Sci Rep. 2023 Oct 3;13:16597. doi: 10.1038/s41598-023-43767-7 (PMC10547781; doi:10.1038/s41598-023-43767-7)
Supplement: Supplementary file 1 — Supplementary Information. [file 41598_2023_43767_MOESM1_ESM.pdf]

Human papillomavirus and prostate cancer: systematic review and meta-analysis

Irina A. Tsydenova, Marina K. Ibragimova, Matvey M. Tsyganov, and Nikolai V. Litviakov

PICOS components

P*opulation: men who have a morphologically confirmed diagnosis of prostate cancer and healthy men for control group.*

I*ntervention (Вмешательство): For the meta-analysis, it was important that the study material was tissue, paraffin blocks or frozen tissue that was clinically validated. All patients had a treatment-naive and prostate-specific antigen (PSA)-positive.*

C*ontrol action:HPV infection in tumour tissue of prostate cancer patients compared to normal prostate tissue of healthy men*

O*utcomes: Reduced incidence of disease in men. Intensify existing HPV vaccination strategies. Identify the association between the presence of HPV and the risk of prostate cancer.*

S*tudies: Compliance with PRISMA items and strict compliance with inclusion criteria. Case-control studies that report sufficient data to calculate risk.*

Supplementary Table 1. Excluded articles

Author, Doi	Year	Country	Exclusion reasons
Korodi et al.<br>Doi:10.1158/1055-9965.EPI-05-0602	2005	Sweden	Blood serum was used as study material
Dillner et al.<br>Doi:10.1002/(sici)1097-0215(19980209)75:4<564::aid-ijc12>3.0.co;2-9	1998	Finland	Blood serum was used as study material
Pascale et al.<br>Doi:10.1155/2013/735843	2013	Italy	Authors used immunohistochemistry, there was no control.
Hayes et al.<br>Doi:10.1016/s0197-2456(00)00101-x	2000	USA	Authors did not use prostate tissue and PCR as method for detection; HPV seropositivity was determined by standard ELISA
Adami et al.	2003	Sweden	Authors did not use prostate tissue and PCR as method for detection; HPV seropositivity was determined by standard ELISA
Masood et al.<br>Doi:10.1097/00007611-199102000-00020	1991	US	Authors used in situ DNA hybridization for human papillomavirus using commercially available biotinylated DNA probes detected by an avidin-biotin peroxidase technique
Zambrano et al.<br>Doi:10.1002/pros.10157	2002	USA	There was no control.
Mokhtari et al.	2013	Iran	Immunohistochemical (IHC) staining was performed for all selected blocks to detect HPV infection.
Korodi et al.<br>Doi:10.1158/1055-9965.EPI-05-0602	2005	Nordic	Blood serum was used as study material. HPV seropositivity was determined by standard ELISA.
Chen et al.<br>Doi:10.1007/s12253-010-9357-4	2011	Australia	Prostate tumour tissue is not used.
Sitas et al.	2007	South Africa	Sera was used as study material. Authors used an anti-HPV IgG enzyme-linked immunosorbent assay (ELISA)
Sutcliffe et al.<br>Doi:10.1158/1055-9965.EPI-07-0134	2007	US	Blood serum was used as study material. HPV-16, HPV-18, and HPV-33 antibody serostatus were assessed by enzyme-based immunoassays.
Hisada et al.<br>Doi:10.1001/jama.283.3.340-a	2000	USA	Blood serum was used as study material.

Rosenblatt et al.	2003	USA	Blood serum was used as study material. Seropositivity for HPV was determined by the standard ELISA assay.
Huang et al.<br>Doi:10.1158%2F1055-9965.EPI-08-0173	2008	USA	Blood serum was used as study material.
Chen et al.<br>Doi:10.1371/journal.pone.0128955	2015	China	Authors used RNA-seq data.
Serth et al.	1999	Germany	Direct PCR sequence analysis
Anwar et al.	1992	Japan	There was no control.
Dennis et al.<br>Doi:10.1158/1055-9965.EPI-08-1167	2009	US	Blood serum was used as study material.
Sutcliffe et al.<br>Doi:10.1158/1055-9965.EPI-09-1080	2010	US	Blood serum was used as study material. HPV seropositivity was determined by standard ELISA.
Hrbacek et al.<br>Doi:10.1186/1471-2407-11-53	2011	Czech	Blood serum was used as study material. Serum samples were analysed by means of enzyme-linked immunosorbent assay
Zhao et al.	2017	China	Blood serum was used as study material.
Mahbobi AI	2016	Iraq	Detection and genotyping of HPV was done by highly sensitive in situ hybridization technique.
Kuczyk et al.<br>Doi:10.1038/sj.pcan.4500448	2000	Germany	Diagnoses wasn't confirmed morphologically.
Atashafrooz et al.	2016	Iran	Diagnoses wasn't confirmed morphologically.
Spinu et al.	2018	Romania	Prostate tumour tissue is not used.
Al-Ahdal et al.<br>Doi:10.1136/sti.72.5.345	1996	Saudi Arabia	Prostate tumour tissue is not used. Diagnoses wasn't confirmed morphologically.
Al-Mahhobi et al.	2011	Iraq	Authors used in situ hybridization for human papillomavirus.
Gazzaz et al.	2009	Kingdom of Saudi Arabia	Authors used HPV DNA HC2 Digene test.
Araujo-Neto et al.<br>Doi:10.1590/1678-4685-GMB-2015-0122	2016	Brazil	There was no control.
Breyer et al.<br>Doi:10.1111/bju.13050	2015	USA	Blood serum was used as study material.
Glenn et al.<br>Doi:10.1186/s13027-017-0157-2	2017	Australia	Authors used only benign prostate hyperplasia later developed prostate cancer.
Rodriguez et al.<br>Doi:10.22034/APJCP.2016.17.11.4863	2016	Mexico	HPV was detected by INNOLiPA HPV.

Wideroff et al.<br>Doi:10.1002/(SICI)1097-0045(199602)28:2<117::AID-PROS7>3.0.CO;2-D	1996	USA	Authors detected HPV by hybridization by dot blot.

Supplementary Table 2. Search strategy

Search	Query
#1	(“Human papillomavirus” OR “human papilloma virus” OR “HPV” OR “Human papillomavirus 16” OR “Human papillomavirus 18” OR “Papillomavirus Infections”)
#2	(“Prostate cancer” OR “prostate tumour” OR “prostate tumour tissue*”)
#3	(“Benign prostatic hyperplasia” OR “BPH” OR “Benign prostatic hypertrophy”)
#4	#1 AND #2<br>(“Human papillomavirus” OR “human papilloma virus” OR “HPV” OR “Human papillomavirus 16” OR “Human papillomavirus 18” OR “Papillomavirus Infections”) AND (“Prostate cancer” OR “prostate tumour” OR “prostate cancer*”)
#5	#1 AND #3<br>(“Human papillomavirus” OR “human papilloma virus” OR “HPV” OR “Human papillomavirus 16” OR “Human papillomavirus 18” OR “Papillomavirus Infections”) AND (“Benign prostatic hyperplasia” OR “BPH” OR “Benign prostatic hypertrophy”)
#6	(“Risk factors” OR “Risk factors of prostate cancer” OR “HPV as a risk factor of prostate cancer” OR “Viral load” OR “Detection of cancer” OR “Biomarkers, Tumour” OR “Prevalence”)
#7	#1 AND #6<br>(“Human papillomavirus” OR “human papilloma virus” OR “HPV” OR “Human papillomavirus 16” OR “Human papillomavirus 18” OR “Papillomavirus Infections”) AND (“Risk factors” OR “Risk factors of prostate cancer” OR “HPV as a risk factor of prostate cancer” OR “Viral load” OR “Detection of cancer” OR “Biomarkers, Tumour” OR “Prevalence”)

*In Scopus, search was limited to “articles”, “reviews”, “conference papers” and “short surveys” using filters.

Supplementary Table 3. Joanna Briggs Institute (JBI) Critical Appraisal Checklist and risk of bias for Case Control Studies

Author, year, country	Were the groups comparable other than the presence of disease in cases or the absence of disease in controls?	Were cases and controls matched appropriately?	Were the same criteria used for identification of cases and controls?	Was exposure measured in a standard, valid and reliable way?	Was exposure measured in the same way for cases and controls?	Were confounding factors identified?	Were strategies to deal with confounding factors stated?	Were outcomes assessed in a standard, valid and reliable way for cases and controls?	Was the exposure period of interest long enough to be meaningful?	Was appropriate statistical analysis used?	**Total**	**Grade**
McNicol and Dodd, 1991, Canada											**5**	*Low*
Ibrahim et al., 1992, US											**5**	*Low*
Suzuki et al., 1996, Japan											**4**	*Low*
Terris and Peehl, 1997, USA											**4**	*Low*
Martinez-Fierro et al., 2010, Mexico											**8**	*High*
Whitaker et al, 2013, Australia											**5**	*Moderate*
Michopoulou et al., 2014, Greece											**6**	*Moderate*
Smelov et al, 2016, Russia											**4**	*Low*
Nahand et al., 2020, Iran											**9**	*High*
Fatemipour et al., 2021, Iran											**9**	*High*
Rotola et al., 1992, Italy											**5**	*Low*
Moyret-Lalle et al., 1995, France											**5**	*Low*
Noda et al., 1998, Japan											**4**	*Low*
Serth et al., 1999, Germany											**6**	*Moderate*
Carozzi et al, 2004, Italy											**7**	*High*
Leiros et al., 2005, Argentina											**6**	*Moderate*
Bergh et al, 2007, Sweden											**7**	*High*
Silverstre et al., 2009, Brazil											**5**	*Low*
Chen et al., 2011, Australia											**5**	*Low*

Aghakhani et al., 2011, Iran											**6**	*Moderate*
Tachezy et al, 2012, Czech Republic											**8**	*High*
Salehi and Hadavi, 2012, Iran											**6**	*Moderate*
Singh et al, 2015, India											**7**	*High*
Huang L et al, 2016, China											**4**	*Low*
Aydin et al., 2017, Turkey											**7**	*High*
Abdolmaleki et al., 2018, Iran											**7**	*High*
Medel-Flores et al., 2018, Mexico											**8**	*High*
**Yes**	**No**				**Unclear**				**Not applicable**			

JBI Critical Appraisal Checklist for case control studies

	Yes	No	Unclear	Not applicable
1. Were the groups comparable other than the presence of disease in cases or the absence of disease in controls?	□	□	□	□
2. Were cases and controls matched appropriately?	□	□	□	□
3. Were the same criteria used for identification of cases and controls?	□	□	□	□
4. Was exposure measured in a standard, valid and reliable way?	□	□	□	□
5. Was exposure measured in the same way for cases and controls?	□	□	□	□
6. Were confounding factors identified?	□	□	□	□
7. Were strategies to deal with confounding factors stated?	□	□	□	□
8. Were outcomes assessed in a standard, valid and reliable way for cases and controls?	□	□	□	□
9. Was the exposure period of interest long enough to be meaningful?	□	□	□	□
10. Was appropriate statistical analysis used?	□	□	□	□

Supplementary Table 4. GRADE: Grading of Recommendations, Assessment, Development and Evaluations

Study name	Certainty assessment					Certainty
	Risk of bias	Inconsistency	Indirectness	Imprecision	Other consideration	
Study design: observational study (case-control studies)						
McNicol and Dodd, 1991, Canada	Serious[1]	Not serious	Not serious	Serious[2]	None	⊕◯◯◯ **Very low**
Ibrahim et al., 1992, US	Serious[1]	Not serious	Not serious	Not serious	None	⊕◯◯◯ **Very low**
Suzuki et al., 1996, Japan	Serious[1]	Not serious	Not serious	Not serious	None	⊕◯◯◯ **Very low**
Terris and Peehl, 1997, USA	Serious[1]	Not serious	Not serious	Not serious	None	⊕◯◯◯ **Very low**
Martinez-Fierro et al., 2010, Mexico	Not serious	Not serious	Not serious	Not serious	None	⊕⊕◯◯ **Low**
Whitaker et al, 2013, Australia	Not serious	Not serious	Not serious	Not serious	None	⊕⊕◯◯ **Low**
Michopoulou et al., 2014, Greece	Not serious	Not serious	Not serious	Not serious	None	⊕⊕◯◯ **Low**
Smelov et al, 2016, Russia	Serious[1]	Not serious	Not serious	Not serious	None	⊕◯◯◯ **Very low**
Nahand et al., 2020, Iran	Not serious	Not serious	Not serious	Not serious	None	⊕⊕◯◯ **Low**
Fatemipour et al., 2021, Iran	Not serious	Not serious	Not serious	Not serious	None	⊕⊕◯◯ **Low**
Rotola et al., 1992, Italy	Serious[1]	Not serious	Not serious	Not serious	None	⊕◯◯◯ **Very low**
Moyret-Lalle et al., 1995, France	Serious[1]	Not serious	Not serious	Not serious	None	⊕◯◯◯ **Very low**
Noda et al., 1998, Japan	Serious[1]	Not serious	Not serious	Not serious	None	⊕◯◯◯ **Very low**
Serth et al., 1999, Germany	Not serious	Not serious	Not serious	Not serious	None	⊕⊕◯◯ **Low**

Carozzi et al, 2004, Italy	Not serious	Not serious	Not serious	Not serious	None	⊕⊕○○ **Low**
Leiros et al., 2005, Argentina	Not serious	Not serious	Not serious	Not serious	None	⊕⊕○○ **Low**
Bergh et al, 2007, Sweden	Not serious	Not serious	Not serious	Not serious	None	⊕⊕○○ **Low**
Silverstre et al., 2009, Brazil	Serious[1]	Not serious	Not serious	Not serious	None	⊕⊕○○ **Low**
Chen et al., 2011, Australia	Serious[1]	Not serious	Not serious	Not serious	None	⊕⊕○○ **Low**
Aghakhani et al., 2011, Iran	Not serious	Not serious	Not serious	Not serious	None	⊕⊕○○ **Low**
Tachezy et al, 2012, Czech Republic	Not serious	Not serious	Not serious	Not serious	None	⊕⊕○○ **Low**
Salehi and Hadavi, 2012, Iran	Not serious	Not serious	Not serious	Not serious	None	⊕⊕○○ **Low**
Singh et al, 2015, India	Not serious	Not serious	Not serious	Not serious	None	⊕⊕○○ **Low**
Huang L et al, 2016, China	Serious[1]	Not serious	Not serious	Not serious	None	⊕○○○ **Very low**
Aydin et al., 2017, Turkey	Not serious	Not serious	Not serious	Not serious	None	⊕⊕○○ **Low**
Abdolmaleki et al., 2018, Iran	Not serious	Not serious	Not serious	Not serious	None	⊕⊕○○ **Low**
Medel-Flores et al., 2018, Mexico	Not serious	Not serious	Not serious	Not serious	Strong association	⊕⊕⊕○ **Moderate**

JBI: Joanna Briggs Institute Critical Appraisal Checklist and risk of bias for Case Control Studies, RR**:** risk ratio

[1]These studies were found having serious risk of bias based on JBI tools.
*The quality for case-control studies of evidence is initially considered as low because the study design itself is observational and lacks randomization.

High quality: Confidence that the true effect lies close to that of the estimate of the effect
Moderate quality: Moderate confidence in the effect estimate. The true effect is likely to be close to the estimate of the effect, but there is a possibility that it is substantially different.
Low quality: Limited confidence in the effect estimate. The true effect may be substantially different from the estimate of the effect
Very low quality: Very little confidence in the effect estimate. The true effect is likely to be substantially different from the estimate of effect
